# Characterizing the molecular epidemiology of *Staphylococcus aureus* across and within fitness facility types

**DOI:** 10.1186/s12879-019-3699-7

**Published:** 2019-01-18

**Authors:** Mark Dalman, Sabana Bhatta, Nagashreyaa Nagajothi, Dipendra Thapaliya, Hailee Olson, Haji Mohammad Naimi, Tara C. Smith

**Affiliations:** 1 Kent State University, College of Podiatric Medicine, 6000 Rockside Woods Blvd. N, Independence, OH 44131 USA; 2 Department of Biostatistics, Environmental Health Sciences and Epidemiology, Kent State University, College of Public Health, Kent, OH USA; 3 Department of Microbiology, Kabul University, Faculty of Pharmacy, Jamal Meena street, Kabul, Afghanistan

**Keywords:** Athletic fitness facilities, *S. aureus*, MRSA, Multi-drug resistant, Northeast Ohio, CrossFit, Gym

## Abstract

**Background:**

*Staphylococcus aureus* is a common bacterium found in the nose and throat of healthy individuals, and presents risk factors for infection and death. We investigated environmental contamination of fitness facilities with *S. aureus* in order to determine molecular types and antibiotic susceptibility profiles of contaminates that may be transmitted to facility patrons.

**Methods:**

Environmental swabs (*n* = 288) were obtained from several fitness facilities (*n* = 16) across Northeast Ohio including cross-fit type facilities (*n* = 4), traditional iron gyms (n = 4), community center-based facilities (*n* = 5), and hospital-associated facilities (*n* = 3). Samples were taken from 18 different surfaces at each facility and were processed within 24 h using typical bacteriological methods. Positive isolates were subjected to antibiotic susceptibility testing and molecular characterization (PVL and *mec*A PCR, and *spa* typing).

**Results:**

The overall prevalence of *S. aureus* on environmental surfaces in the fitness facilities was 38.2% (110/288). The most commonly colonized surfaces were the weight ball (62.5%), cable driven curl bar, and CrossFit box (62.5%), as well as the weight plates (56.3%) and treadmill handle (50%). Interestingly, the bathroom levers and door handles were the least contaminated surfaces in both male and female restroom facilities (18.8%). Community gyms (40.0%) had the highest contamination prevalence among sampled surfaces with CrossFit (38.9%), traditional gyms (38.9%), and hospital associated (33.3%) contaminated less frequently, though the differences were not significant (*p* = 0.875). The top *spa* types found overall were t008 (12.7%), t267 (10.0%), t160, t282, t338 (all at 5.5%), t012 and t442 (4.5%), and t002 (3.6%). t008 and t002 was found in all fitness facility types accept Crossfit, with t267 (25%), t548, t377, t189 (all 10.7%) the top *spa* types found within crossfit. All samples were resistant to benzylpenicillin, with community centers having significantly more strains resistant to oxacillin (52.8%), erythromycin (47%), clindamycin (36%), and ciprofloxacin (19%). Overall, 36.3% of isolates were multidrug resistant.

**Conclusions:**

Our pilot study indicates that all facility types were contaminated by *S. aureus* and MRSA, and that additional studies are needed to characterize the microbiome structure of surfaces at different fitness facility types and the patrons at these facilities.

## Background

*Staphylococcus aureus* is an opportunistic pathogen that colonizes asymptomatically the skin and nares of approximately 1 in 3 people worldwide [1, 2]. *S. aureus* is found in hospital settings, and with the emergence of antibiotic resistance, can cause life threatening infections. Recent studies have suggested that, in the United States, over 80,000 invasive infections and 11,000 yearly deaths are attributed to methicillin-resistant *S. aureus* (MRSA) [3]. While the number of invasive MRSA infections has declined slowly over the past decade, our group has identified reservoirs of MSSA (methicillin-susceptible *S. aureus*) and MRSA in humans, animals, and the environment [4–11]. Although colonization with *S. aureus* is usually asymptomatic, it is a risk factor for active infection [12] and enhances the ability of an individual to transmit *S. aureus* to fomite surfaces [13].

One location that has been associated with an increased risk of both infection and colonization with *S. aureus* is the athletic fitness facility [14, 15]. Over 50 million individuals in the United States support approximately 30,000 fitness facilities, and the number continues to rise [14]. MRSA infections have been linked to contamination of athletic facilities due to shared exercise equipment [15] and towels [16]. Facility-associated MRSA infections have also been documented in both professional and collegiate athletic teams [17–19], and even in high school athletes [20, 21]. The link between fitness facilities and contaminated surfaces is unclear [22], but Markley and colleagues [15] found that approximately 10% of gym surfaces were contaminated with *S. aureus* and these contaminated fomites may serve to spread *S. aureus*. However, not all fitness facility types and surfaces may warrant the same scrutiny.

In the present study, we assessed the prevalence of *S. aureus* and MRSA across several fitness facility types to characterize the microbial environment and potential for *S. aureus* transmission, to identify potential risks associated with certain areas, environmental conditions, or types of exercise equipment, and to provide a more inclusive study addressing exercise facility type as a facilitator of *S. aureus* transmission.

## Methods

### Facility sampling

We performed point prevalence microbiological surveys at 16 fitness facilities in Northeast Ohio. The facilities were convenience-sampled with the surfaces selected as the most commonly hand-touched areas in the building. Environmental swabs were obtained from CrossFit type facilities (*n* = 4), traditional free weight gyms (*n* = 4), community center facilities (*n* = 5), and hospital –associated facilities (*n* = 3) with the written permission of the owners. Traditional free weight gyms only have weights for lifting and do not offer any other services such as a pool or daycare. Community center facilities were usually larger facilities with a wide range of services from yoga, gyms for basketball or volleyball, swimming pools, daycare services, rock climbing walls, and food preparation. Hospital-associated facilities were linked to a hospital or clinic and had weights, swimming pool, and guided care for rehabilitation purposes, in addition to providing membership access to the general public. CrossFit facilities involved highly intensive, varied movement exercises covering several sports movements, and were required to self-identify as a CrossFit facility for inclusion purposes.

The sampling technique of surfaces was as previously described [7]. Briefly, a sterile Swiffer® cloth was used to wipe down a 9 square inch environmental surface for 10 s using a new set of gloves for each surface as to reduce cross contamination. Samples were then placed in a labeled, individual sterile Whirl-Pak™ bag (Nasco, Fort Atkinson, WI) and stored on ice until returning to the lab for processing within 3 h of acquisition.

### Bacterial culture and identification

Samples were processed as previously described [23]. Samples were reconstituted in 50 mL of sterile 0.1% peptone broth and massaged for 25 s to enhance bacterial recovery. Subsequently, this peptone solution was transferred to a 50 mL aliquot of sterile, 2X Baird Parker Broth (BPB) solution with tellurite enrichment (Sigma products-Sigma Aldrich, St. Louis, MO) and allowed to incubate at 37 °C for 24 h. After incubation, 1 μL inoculums were streaked onto Baird Parker Agar (BPA) with EY tellurite enrichment and selective MRSA agar plates (BBL CHROMagar MRSA, Becton, Dickinson and Company), and allowed to incubate for 48 h at 37 °C. Potential *S. aureus* colonies (black colonies) and presumptive MRSA colonies (mauve colonies on CHROMagar) were then plated on Columbia colistin- nalidixic acid agar with 5% sheep’s blood (CNA; Ramel). Plated CNA were allowed to grow at 37 °C for 24 h. Colonies were confirmed using a series of biochemical assays including: catalase, coagulase, and *S. aureus* latex agglutination (Pastorex Staph-Plus, Bio-Rad, Hercules, CA). Confirmed *S. aureus* isolates were stored at − 80 °C with a single colony used for antibiotic susceptibility testing (AST) and subsequent molecular analyses.

### Molecular characterization

Positive *S. aureus* isolate genomic DNA was isolated using Wizard Genomic DNA preparation kit (Promega, Madison, WI). Polymerase chain reaction (PCR) was used to amplify the presence of methicillin resistance gene (*mec*A) and PVL genes (*luk*S, *luk*F) [24, 25]. Furthermore, Staphylococcus protein A (*spa*; FOR 5’-GAACAA-CGTAACGGCTTCATCC-3′ and REV 5’-CAGCAGTAGTGCCGTTTGCCT) was used for molecular typing [26–28]. Ridom StaphType software was used to assign *spa* types (v2.2.1; Ridom GmbH, Wurzburg, Germany). The Based upon Repeat Pattern (BURP) algorithm was used to group *spa* types based on their genetic proximity [29], as well as Bionumerics software (version 7.6.2). Only *spa* typing was conducted, since previous studies have found high congruence and discriminatory power compared to MLST sequence data [28, 30, 31]. A positive (USA300) and negative control were used for all biochemical and molecular assays.

### Antimicrobial susceptibility testing (AST)

All *S. aureus* isolates were subjected to AST by VITEK 2 system (bioMerieux, Durham, NC; Version R06.01) using AST-GP71 cards according to manufacturer’s and Clinical Laboratory Standards Institute Standards (CLSI, 2012). A (0.5–0.63 OD) bacterial suspension in 0.45% saline was prepared for each sample tested. AST-GP71 cards test for: benzylpenicillin, oxacillin, tetracycline, erythromycin, ciprofloxacin, moxifloxacin, minocycline, clindamycin, trimethoprim-sulfamethoxazole, quinupristin/ dalfopristin, gentamicin, levofloxacin, linezolid, daptomycin, vancomycin, rifampin, tigecycline, and nitrofurantoin. Resistance to ≥3 class of antibiotics was considered as multi-drug resistant (MDR) [32].

### Environmental factors

Temperature and relative humidity measurements were collected at all locations at a central point away from any external door or HVAC vent. Temperature and relative humidity measurements were collected at the end of sampling (~ 45–60 min) to ensure that the temperature and relative humidity were indicative of the gym facility, and not a carryover from transport or previous location. Total patron membership numbers and cleaning regimens were also collected via a self-reported questionnaire.

### Statistical analysis

Association of variables was tested by Pearson’s Chi-square test in addition to Fisher’s exact test for outcome. For all analyses, P was set at 0.05 with all tests carried out using SAS software (Ver. 9.3, SAS Institute Inc., Cary, NC). Minimum spanning tree was conducted using Bionumerics software (7.6.2).

## Results

### Prevalence of *S. aureus*

**Fig. 1:**
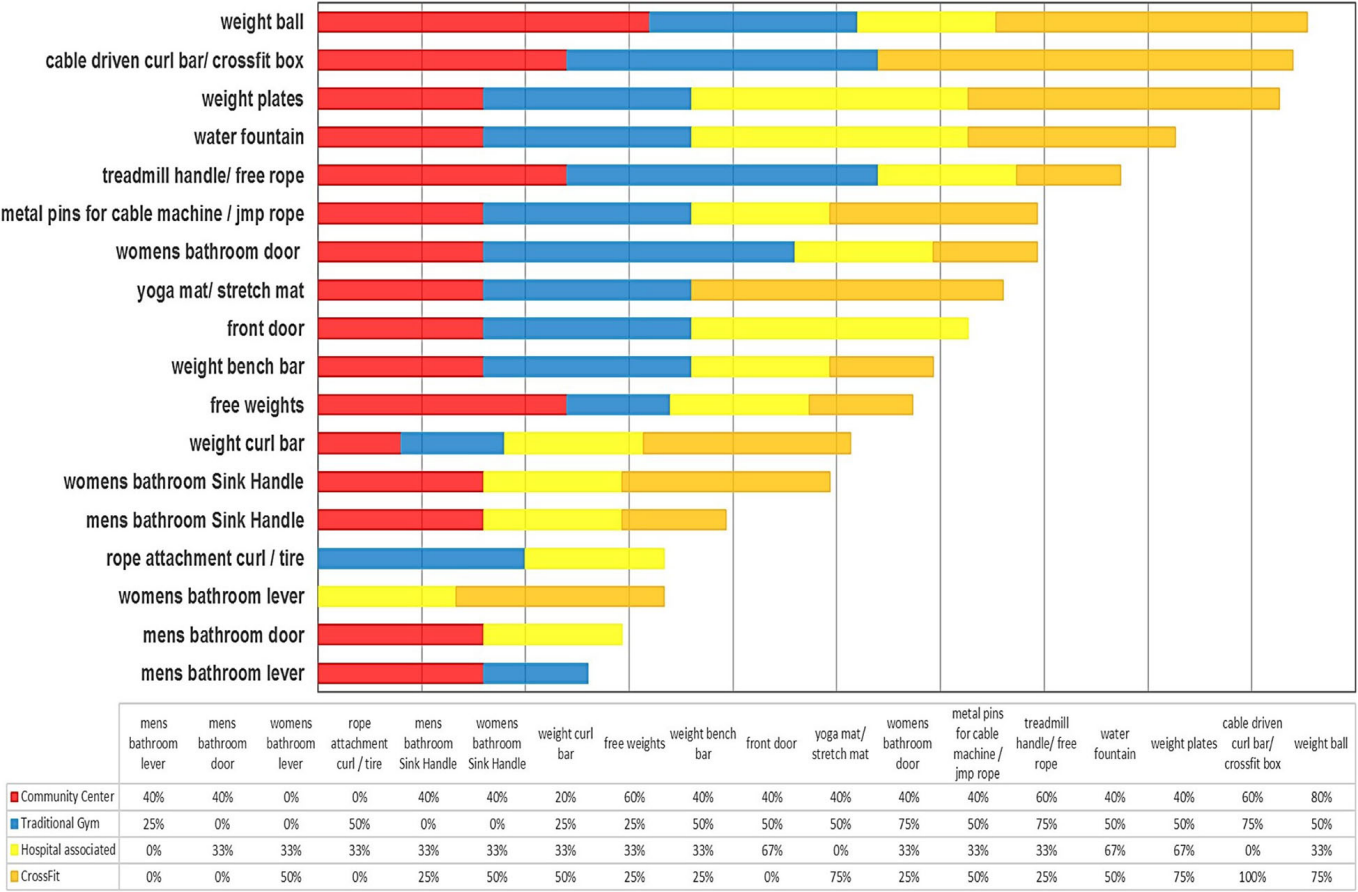
*S. aureus* percent contamination for each fitness facility and surface type

A total of 288 environmental samples (fitness facility surface samples) were collected from 16 fitness facilities in Northeast Ohio. A total of 110 sites were identified as *S. aureus*-positive. The overall prevalence of *S. aureus* for all locations was 38.2% (110/288) with a prevalence of 26.7% (77/ 288; ± 1.65% SE) and 11.5% (33/ 288; ± 3.95% SE) for MSSA and MRSA respectively (Table 1). There was an average of 27.5 ± 3.69 (Mean ± SE) positive isolates per site type. Table 1 shows the distribution of *S. aureus* across multiple fitness facility types sampled. We found similar *S. aureus* contamination across fitness facilities buildings (community: 40.0%, 36/90; traditional: 38.9%, 28/72; CrossFit: 38.9%, 28/72; and hospital-associated: 33.3%, 18/54). The prevalence of MSSA was significantly higher compared to MRSA (*p* = 0.024), while the total number of contaminated surfaces was not significantly different across fitness facility types (Table 1; *p* = 0.875). The highest prevalence of *S. aureus* was observed on the weight ball (62.5%, 10/16) and cable-driven curl bar/ CrossFit box (62.5%, 10/16), followed by weight plates (56.3%, 9/16), treadmill handle/free rope (50.0%, 8/16), and water fountain (50.0%, 8/16) (Fig. 1). Interestingly, based on *mec*A presence, MRSA contamination was higher in community-associated fitness facilities (52.8%, 19/36) compared to hospital-associated (5.56%, 1/18), CrossFit (14.3%, 4/28), and traditional fitness facilities (32.1%, 9/28) (Table 1; *p* = 0.001).

**Table 1:**
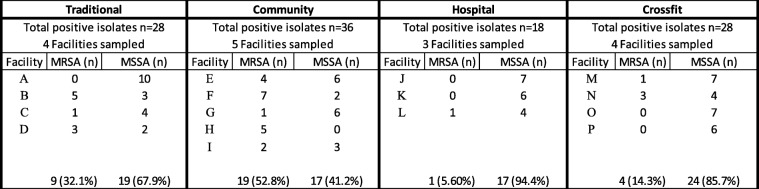
Prevalence of *S. aureus* (MRSA and MSSA) by fitness facility type

MRSA = Methicillin resistant *S. aureus*. MSSA = Methicillin susceptible *S. aureus.*

Under facility type (Traditional, Community, Hospital, and Crossfit), each facility (A through P) had 18 samples taken and only positive MRSA or MSSA are reported here.

Facilities A through P represent unique facility addresses.

### Molecular characterization of *S. aureus* isolates

**Fig. 2:**
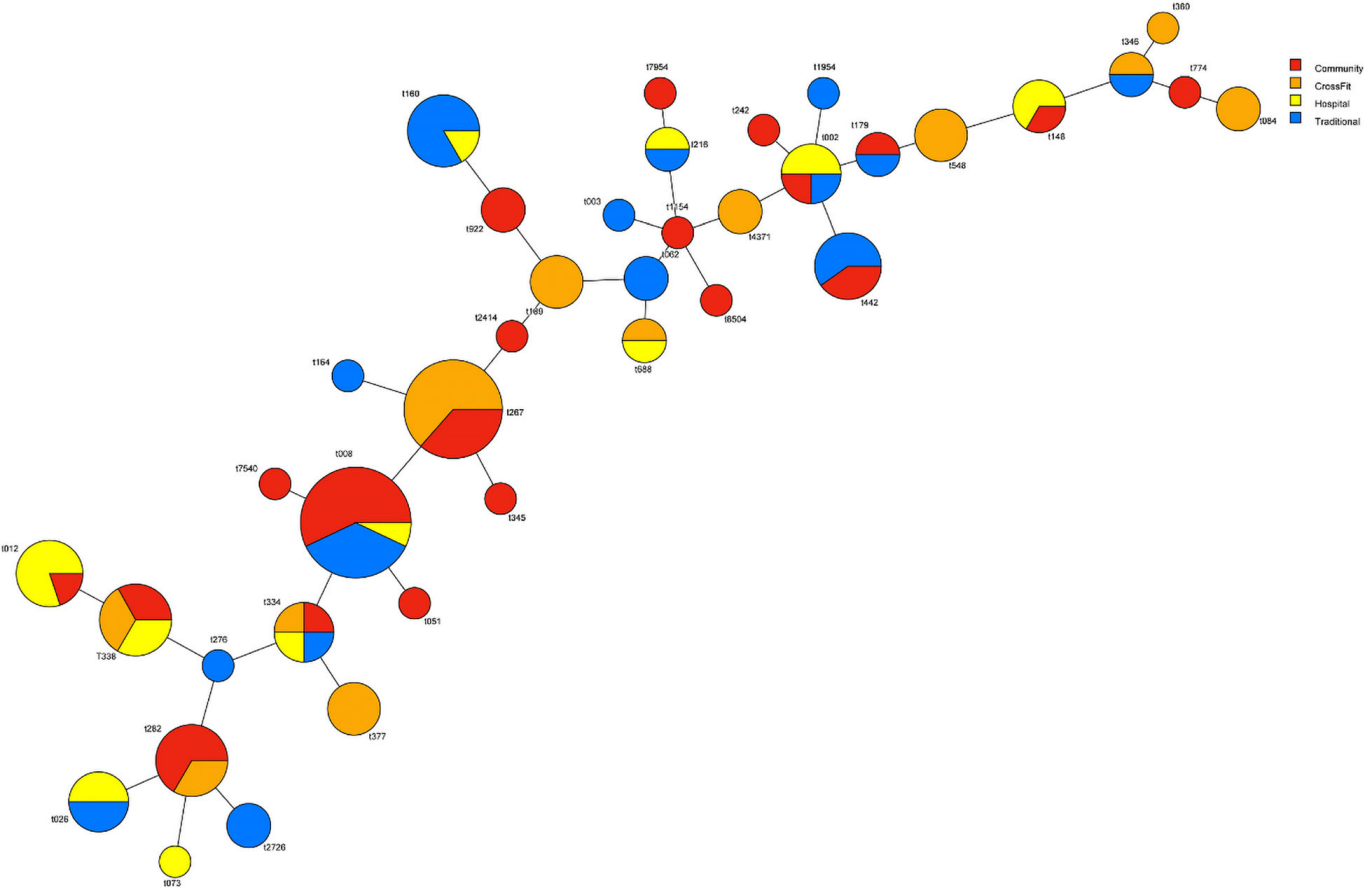
BURP clustering of *spa* typing from *S. aureus* isolates. Color represents fitness facility type with percent of circle equal to percent total of *spa* type in relation to total positive *S. aureus* isolates identified

Molecular typing of the *spa* gene was performed on all confirmed *S. aureus* isolates, in addition to examination of presence of the *mec*A and PVL genes. A total of 38 unique *spa* types were identified among 110 isolates with the most common 14 *spa* types present in at least three or more surfaces. The most common *spa* type present was t008 (12.7%; 14/110), followed by t267 (10.0%; 11/110), t160, t282, t338 (all at 5.45%; 6/110), t012, t442 (both at 4.55%; 5/110), t002, t026, t334 (all at 3.64%; 4/110), t148, t189, t377, and t548 (all at 2.73%; 3/110) (Table 2; Fig. 2). Of 33 MRSA isolates, 33.3% were t008. The most common *spa* types found in each fitness type were t267 (25.0%; 7/28; CrossFit), t012 (22.2%; 4/18; hospital- associated), t008 (22.2%; 8/36; community center), and t008 and t016 were tied for the most common in the traditional free-weight gym (17.9%; 5/28).

**Table 2:**
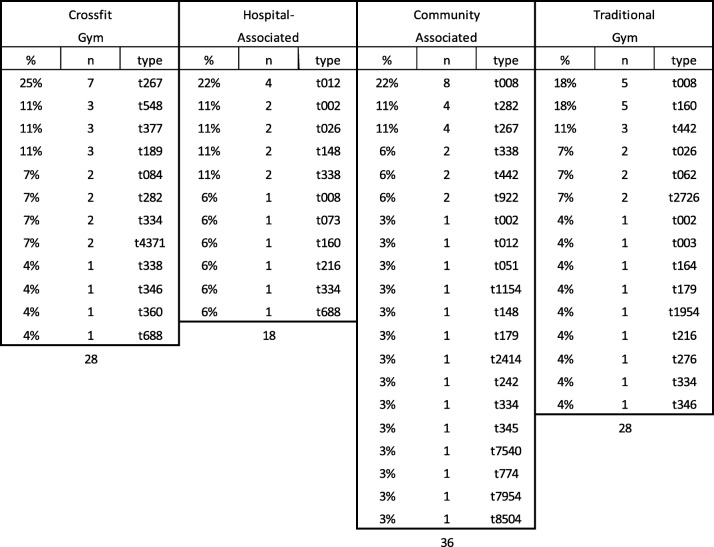
*S. aureus spa* typing by fitness facility location

The prevalence of the *mec*A gene among *S. aureus* isolates was 30.0% (33/110). MRSA was isolated from 14.3% (4/28) of isolates in CrossFit facilitates, 5.6% (1/18) of hospital-associated facilities, 52.8% (19/36) of community facilities, and 32.1% (9/28) of traditional gym facilities (Table 2). There was a significant difference observed between fitness facility types (*p* = 0.001). There were only three isolates (2.7%; 3/110) that were PVL-positive and were correspondingly found only in community facilities.

### Antibiotic susceptibility profile

**Fig. 3:**
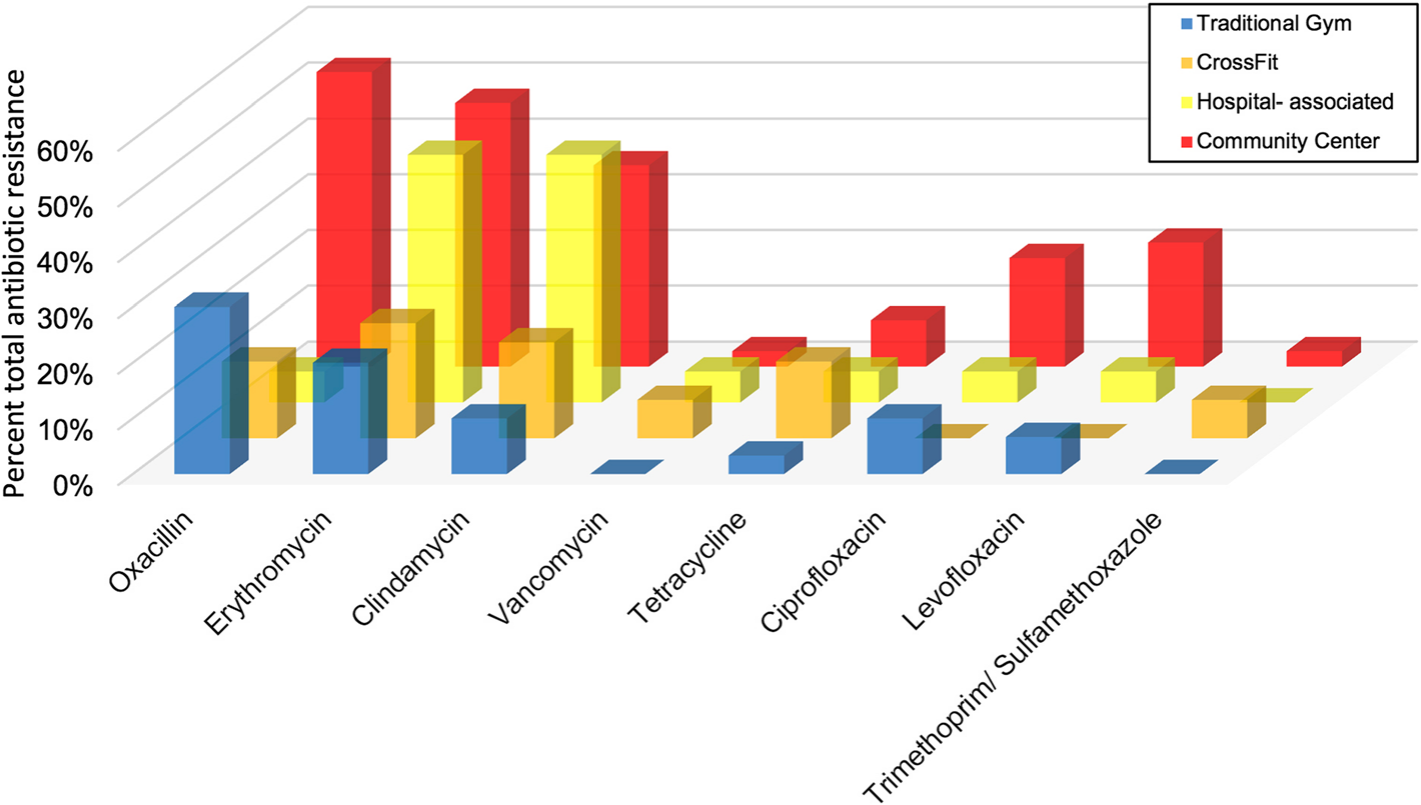
Percent total antibiotic resistance of positive *S. aureus* isolates by fitness facility location. *All vancomycin resistance is intermediate

All *S. aureus* isolates were subjected to antibiotic susceptibility testing. Cumulatively, 37 isolates (33.6%) were resistant to erythromycin, 33 (30.0%) were resistant to oxacillin, 29 (26.4%) were resistant to clindamycin, 9 (8.2%) were resistant to tetracycline, 11 (10.0%) were resistant to ciprofloxacin, 11 (10.0%) were resistant to levofloxacin, and 4 (3.6%) were resistant to minocycline (Fig. 3). Forty isolates (36.3%) were MDR- *S. aureus.* The 4 isolates that demonstrated intermediate resistance to vancomycin were found within community (1), CrossFit (2), and hospital (1) -associated facilities. Community-associated facilities had significantly more oxacillin (52.8%; 19/36; *p* = 0.001), levofloxacin (22.2%; 8/36; *p* = 0.021), clindamycin (36.1%; 13/36; *p* = 0.025), erythromycin (47.2%; 17/36; *p* = 0.054), and ciprofloxacin (19.4%; 7/36; *p* = 0.056) resistant strains compared to hospital, crossfit, and traditional facility types.

### Environmental factors

Temperature and relative humidity measurements were collected at all locations, as were patron membership numbers. The mean facility temperature across all facilities was 21.5 °C (70.7 °F ± 2.4) with a range of 18.3 to 23.3 °C (Data not shown; *p* = 0.156). The average relative humidity was 47.6% ±3.8 with no significant difference between facilities (Data not shown; *p* = 0.708). Patron membership was highest in community centers (7496 ± 4327), with hospital associated facilities at 2400 ± 1053, traditional gyms at 1350 ± 724, and CrossFit at 103 ± 27 (Mean ± SE). However, the patronage difference was not statistically significant across facilities sampled (Data not shown; *p* = 0.22). All facilities provided access to hand sanitizer stations except for 2 CrossFit facilities (50%) and 1 community center (20%).

## Discussion

This study examined the prevalence and molecular characteristics of *S. aureus* and MRSA sampled from 288 gym surfaces collected from 16 gyms across four different types of gym facilities in Northeast Ohio. From 288 samples, 110 were positive *S. aureus* isolates and we had a 26.7% (77/288) and 11.5% (33/288) recovery of *S. aureus* and MRSA, respectively. We found similar *S. aureus* prevalence across community, traditional, CrossFit, and hospital-associated fitness facilities (40.0% vs 38.9% vs 38.9% vs 33.3%, respectively) (Table 1). However, our prevalence rates were higher than those identified previously [15–21]. The prevalence rate difference may be attributed to fitness facility and/or patron type. As athletes and athletic personnel generally have a significantly higher incidence of both infections and over a ten-fold higher number of antibiotic prescriptions per year compared to the general public, many infections may remain unmonitored or unrecognized [18, 33, 34]. Despite their continual occurrence, there has been little effort to identify and monitor contaminated surfaces and the role they may play in transmission, until recently [35–39]. Though our study found similar rates of contamination across all fitness facilities that mirror common human carriage rates, the incidence of both *S. aureus* and MRSA is higher in our environmental contamination study than what has been observed in the literature, such as in school settings, playgrounds, and beaches [4–8, 15, 22, 40]. Despite other environmental contamination studies, studies of fitness facilities have received attention only recently.

For example, Ryan and colleagues [22] found zero presence of *S. aureus* in gym facility surfaces suggesting that all transmission was entirely via-person to person contact or at least ruling out that gyms were reservoirs of *S. aureus.* Markley and colleagues [15] sampled 16 different surface types at one large community center and found that 10% (10/99) of samples were contaminated with MSSA only. Due to the significantly limited size and scope of the study (one facility type was examined), their reported incidence may be underestimated. Prior to these studies, 5 players (9%) on the 2003 St. Louis Rams football team were found to have MRSA infections [18]. Interestingly, they found zero nasal carriers and/or environmental reservoirs of MRSA, but did grow MSSA from whirlpool water and a gel-applicator stick used for taping ankles, suggesting that fomite surfaces have the potential to harbor and transmit *S. aureus*. Although Ryan and colleagues [22] surveyed three facilities (college, high school, and private gyms) before and after cleaning regiments, they found zero presence of MSSA and MRSA from their 240 samples. Their results may be attributed to differences in bacterial isolation and cultivation. Almost 40% of the population are carriers of *S. aureus* [41], thus, it is surprising that these studies found very few contaminated surfaces, while contamination in a hospital setting is sufficiently ubiquitous to sound alarm [42–46]. For example, a hospital study found that 76% of skin and soft tissue infections were of *S. aureus* etiology and 59% of those were attributable to MRSA [47]. Of those presenting MRSA, 99% of isolates were community-associated (CA-MRSA), pointing to acquisition of MRSA strains from outside of the hospital setting, such as a gym facility. Likewise, hospital ward high touch surfaces areas were highly contaminated with *S. aureus* concentration increasing by almost 80% over a 4-h period despite the use of hypochlorite. The addition of Quaternary Ammonium Compound surfactants (QAC) did drastically decontaminate surfaces to almost 10% of their original bacterial load count, pointing to potential bacterial decontamination strategies to reduce transmission [46]. Additionally, through the use of sequencing techniques, small amounts of biomass collected from gym facilities were sufficient to identify community bacteria, as well as staphylococcal species present on athletic surfaces in the gym, with the composition modulated by interacting with human skin [48]. Though our study did not track personnel or patron *S. aureus* carriage or microbiome composition, provenance of contamination will be key for future studies addressing the movement, transmission, and potential antibiotic-resistant reservoirs of fitness facilities.

The increased prevalence of *S. aureus* on fitness facility surfaces may also be a result of environmental co-evolution. As *S. aureus* can tolerate high osmolarity stress (high saline environments), the production of sweat at gym facilities can even be extrapolated to other high intensity situations such as war and combat, which may select for *Staphylococcal* species in the environment or individual [49]. With ease of horizontal gene transfer, the acquisition of antibiotic resistance may be enhanced as a result. The incidence of MRSA in athletes is almost triple what is observed in the general population. Thus, it is not surprising that we observed higher contamination rates on gym surfaces than other surfaces [50].

We found a total of 38 unique *spa* types with t008 (14; 12.7%) and t267 (11; 10.0%) being the most common (Table 2; Fig. 2). Interestingly, some loosely identified livestock associated strains such as t548 and t338 were found in Crossfit, community, and hospital-associated strains but not in strains isolated from traditional gyms (Fig. 2). t548 is associated to upper Midwestern and northwestern regions of the US, including Ohio. Since it was found in hospital associated gyms, the line between the original provenance of *S. aureus* strains and their site of contamination is becoming increasingly fluid. Approximately 8.2% (9/110) of strains were categorized as livestock-associated. A total of 6 isolates were t338 and were found in hospital, Crossfit, and community fitness facilities. Three isolates (2.7%) were t548 and found solely within Crossfit facilities. Additionally, t012 was the most common strain type found in hospital-associated facilities (4; 22.0%) and community fitness centers (1; 3.0%), but not traditional or Crossfit facilities. t012 is known to be less prevalent as the age of the individual increases [51, 52]. However, its increased incidence in hospital- associated facilities may be a result of rehabilitation of both older and younger patients. Conversely, t002 was found in hospital-associated (2; 11.0%), community (1; 3.0%), and traditional facilities (1; 4.0%). t002 is often found in nursing homes and in older patients [53]. As such, the hospital-associated facilities also had strains associated with older patients. These results suggest that age demographics may play a significant role in strain isolates found in various fitness facilities. We found t002 on the weight ball and weight bench bars in hospital, traditional, and community facilities. As it has the possibility to be present in cases of bacteremia [52, 54], it is important to be careful in regards to lifting weights with any cuts present, which could contaminate gym equipment.

Looking across gym facilities, Crossfit had the most diverse range of *spa* types (Table 2; Fig. 2). Of the 10 *spa* types found in Crossfit facilities, 6 were community-associated (t267, t377, t084, t282, t334, and t4371), two have been found in livestock (t548 and t338), and two have been associated in hospital settings (t189 and t346). Though other facilities sampled had some similar variations of these associations, none were as diverse as Crossfit types. This may be a result of the comparative lack of lifting machine structure in Crossfit gyms and/ or the wide range of people that attend them [55, 56]. Furthermore, if common cleaning regimens are not followed, high hand-touch surfaces may harbor and easily disseminate pathogenic, antibiotic resistant bacteria to other people via hand contact, as hands are recognized as a primary mode of transmission of many diseases (Fig. 3), [38, 45, 57].

To the best of our knowledge, this is the first study to evaluate systematically different gym facility types for contamination and molecular typing of *S. aureus*. The strength of our study resides within our large sample size both across fitness facility types, as well as across fitness surfaces sampled. In addition, we also carried out molecular genotyping of *S. aureus* isolates, further strengthening our epidemiological study of *S. aureus* contamination on athletic gym surfaces. However, there are limitations to our study that included convenience sampling in Northeast Ohio fitness facilities only. In addition, we only sampled surfaces at one point in time, we did not sample surfaces after a cleaning regimen, and we did not sample and type isolates found on patrons and facility employees. Future studies should evaluate the patron microbiome, as well as the collective microbiota impact on fomite contamination and transmission. Our results indicate not only the presence of putatively dangerous isolates of *S. aureus,* but also that increased cleaning regimens and enhanced hygiene practices should be followed in fitness facilities as is practiced in the hospital or work place.

## Conclusions

Of 288 surfaces swabbed from 16 different facilities (traditional gyms, community centers, hospital-associated facilities, and CrossFit facilities), 38.2% (110/288) of surfaces were positive for *S. aureus.* 30.0% of all isolates were mecA-positive with community-associated fitness facilities containing the most mecA (17.3%) compared to traditional, CrossFit, and hospital-associated fitness centers (8.20, 3.64 and 0.91%, respectively). t008 was the most common spa type present across all gym facility types. All fitness locations were contaminated with both *S. aureus* and MRSA, and 36.4% of all positive isolates were multidrug-resistant.

## Abbreviations

AST: antibiotic susceptibility testing
BURP: Based upon Repeat Pattern
CA-MRSA: community-associated Methicillin resistant *Staphylococcus aureus*
HVAC: Heating, ventilation, and air conditioning
MDR: multi-drug resistant
MLST: Multilocus sequence typing
MRSA: Methicillin resistant *Staphylococcus aureus*
MSSA: methicillin-susceptible *S. aureus*
PVL: Panton–Valentine leukocidin PCR Polymerase chain reaction
QAC: Quaternary Ammonium Compound surfactants
